# Concealed Foreign Body Shrouding Airway Mimicking Mass Causing Extubation Failure, Hypoxia, and Stridor

**DOI:** 10.7759/cureus.26338

**Published:** 2022-06-26

**Authors:** Khizar Hamid, Swaminathan Perinkulam Sathyanarayanan, Joe Devasahayam

**Affiliations:** 1 Internal Medicine, University of South Dakota Sanford School of Medicine, Sioux Falls, USA; 2 Pulmonary Critical Care, Avera McKennan Hospital and University Health Center, Sioux Falls, USA

**Keywords:** critical care, extubation failure, airway, icu, foreign body, extubation, intubation

## Abstract

Foreign body (FB) aspiration can present with acute life-threatening asphyxiation to recurrent infections with lung damage. Although most esophageal FBs pass spontaneously, sharp ones can get embedded requiring treatment. Tracheobronchial FBs and hypopharyngeal FBs are occasionally seen as well. We present a case of an oropharyngeal FB presenting with signs of stroke, pulmonary embolism, pulseless, and causing airway compression and extubation failure. Old age and neurocognitive disability are important predisposing factors of FB airway obstruction (FBAO), with food being the most common cause. The classic triad of cough, dyspnea, and cyanosis is seen in only a small percentage of patients with FBAO. Laryngeal edema, soft tissue collapse, and laryngospasm are among the common causes of upper airway obstruction and extubation failure. Laryngeal traumatism that can occur during emergency intubations can cause post-extubation stridor that can be treated with corticosteroids. Dentures and blood have been reported to cause post-extubation complications but oropharyngeal FB causing airway compression and leading to extubation failure has not been reported before. We recommend FB to be considered in the differential diagnosis of immediate hypoxia and extubation failure regardless of the history of a witnessed aspiration event as it is an easily fixable cause and can be missed in the initial history of presentation. A high degree of suspicion for this should be maintained as it is easy to miss. Computed tomography of the neck can aid in the diagnosis.

## Introduction

Foreign body aspiration (FBA) is an important problem in the United States with presentations ranging from acute life-threatening asphyxiation to recurrent infections with lung damage [1]. The physiological response to FBA is cough; however, ineffective cough usually requires other interventions such as abdominal thrusts, chest thrusts, and back blows [2]. Most esophageal foreign bodies (FBs) pass spontaneously, but sharp ones such as fish bones can get embedded requiring treatment. The preferred method of treatment of such FBs is the endoscopic approach [3]. Tracheobronchial FBA in adults is occasionally seen and can be successfully treated with flexible and rigid bronchoscopy [4]. Incidence of hypopharyngeal FB including food materials and dentures has been reported in the literature [5]. We present an unusual case of an FBA that presented with symptoms of stroke, mimicked a laryngeal mass, and had an associated complication of pulmonary embolism (PE). To the best of our knowledge, this is the first case of food material lodging in the oropharynx causing airway compression and resulting in extubation failure.

## Case presentation

An 88-year-old man, with a medical history of atrial fibrillation on coumadin and metoprolol, hyperlipidemia, and bladder cancer status post-resection, presented after suddenly slumping over at a restaurant while having dinner. Witnesses present at the scene provided immediate care to the patient. He was not verbal, only able to respond by nodding his head, and unable to move the right side of his body. He was transported to the local emergency department and on route became pulseless and underwent cardiopulmonary resuscitation (CPR) with orotracheal intubation, achieving a return of spontaneous circulation in 16 minutes. Post-intubation, bloody secretions were noted in his endotracheal tube (ETT). His labs at presentation were unremarkable except for an elevated blood alcohol level (Table 1).

**Table 1:** Labs at presentation.

Lab	Value
Hemoglobin	14.9 g/dL
White blood cell	12.7 K/µL
Platelets	156 K/µL
Sodium	135 mmol/L
Potassium	4.9 mmol/L
Chloride	105 mmol/L
Bicarbonate	19 mmol/L
Blood urea nitrogen	0.8 mg/dL
Creatinine	1/1 mg/dL
Lactic acid	3.5 mmol/L
Troponin I	0.08 mg/dL
Aspartate aminotransferase	61 U/L
Alanine aminotransferase	54 U/L
Alkaline phosphatase	58 U/L
Blood ethyl alcohol level	107 mg/dL

**Figure 1:**
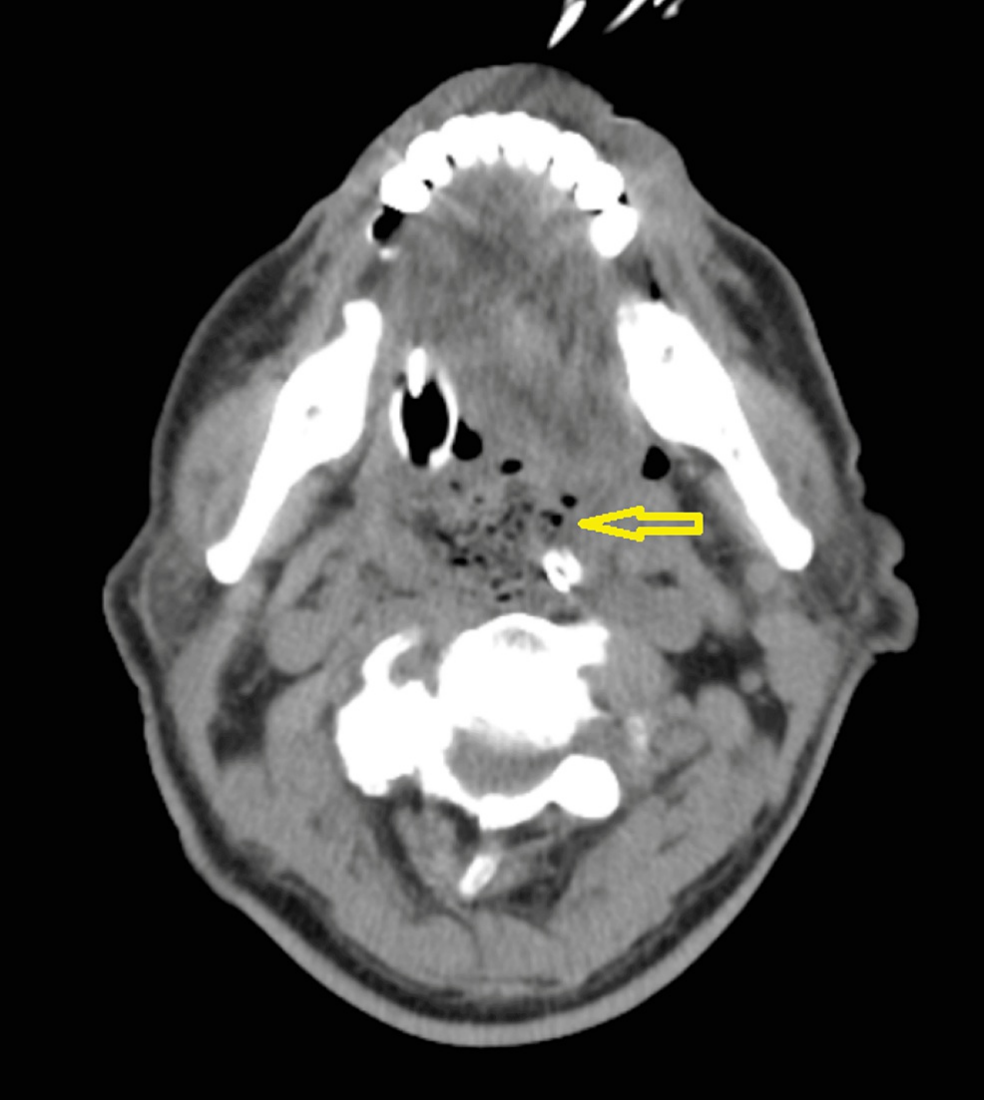
Computed tomography scan of the neck. Transverse section showing 3.1 × 3.3 cm abnormal soft tissue density and air density opacifying the oral pharyngeal and hypopharyngeal airway.

**Figure 2:**
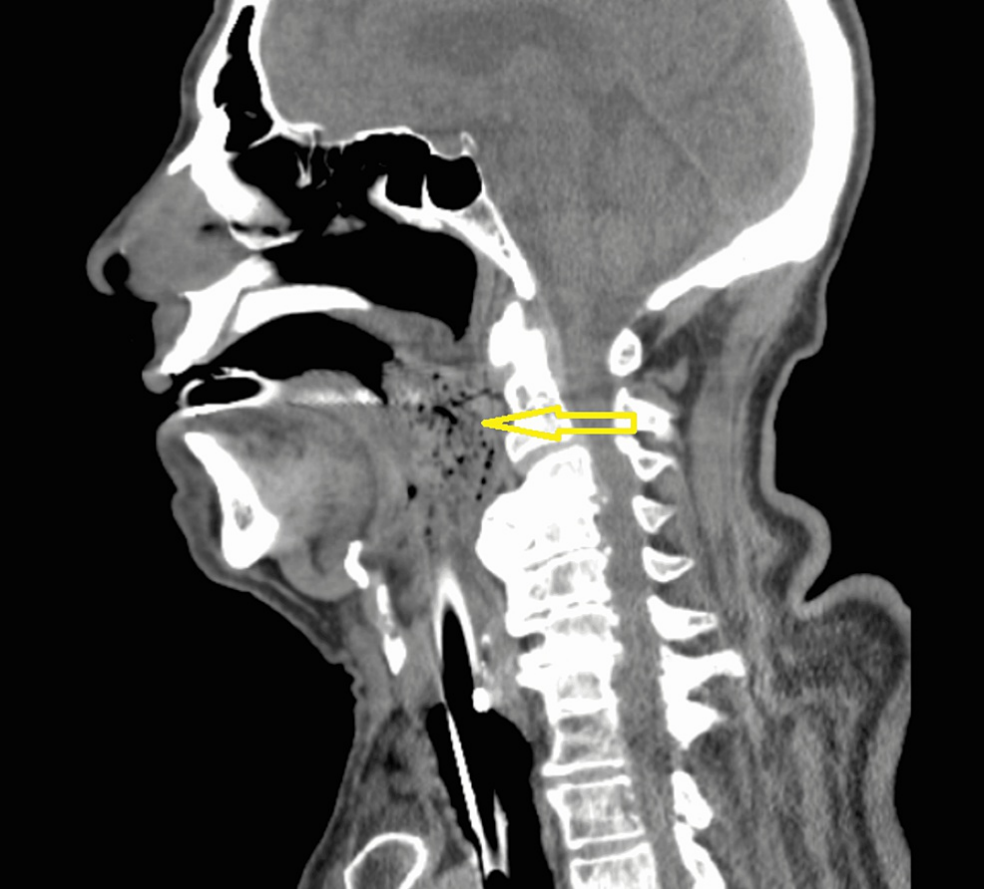
Computed tomography scan of the neck. Sagittal section showing 5.3 cm craniocaudal abnormal material.

Blood cultures and tracheal aspirate culture were negative. Computed tomography (CT) of the head, magnetic resonance imaging (MRI) of the brain, and CT angiogram (CTA) of the head and neck excluded any new stroke, vascular compromise, space-occupying lesion, or oropharyngeal FB. Bronchoscopy and bronchoalveolar lavage cultures were done to evaluate his bloody ETT secretions and excluded any hemorrhage, foreign body, or infection. An echocardiogram was obtained revealing an ejection fraction of 60-65%, borderline elevated left heart filling pressures and dilated right atrium, moderate to severely dilated right ventricle (RV) chamber size, and moderate to severely reduced RV systolic function. This was followed by a CTA of the chest which revealed a left-sided PE with multifocal pneumonia and bilateral pleural effusions resulting in the initiation of treatment with anticoagulation and antibiotics. The next day he was doing remarkably well. He was following commands, responding to questions off sedation, and had complete resolution of his neurological deficit. Extubation was done, and immediately after, he became hypoxic with respiratory distress, cyanosis, and stridorous breath sounds leading to emergency re-intubation. Extubation failure was presumed to be from upper airway edema from his previous intubation during CPR, and he was started on a course of dexamethasone. A CT of the neck was obtained because of stridor identifying abnormal findings of mixed air and soft tissue density nearly occluding the hypopharynx and the oropharyngeal airway (Figures 1, 2). Otolaryngology was consulted and they performed an extensive neck examination and removed a large piece of steak from his oropharynx. Following this, extubation was attempted which was successful.

## Discussion

Foreign body airway obstruction (FBAO) is a universal problem and causes around 5,200 deaths in the United States annually. Old age and neurocognitive disability are common predisposing factors, with food being the most common cause of airway obstruction and death. Meat is the most common esophageal FB seen in Western countries [2,3]. Though our patient did not have any predisposing neurocognitive problems, his old age and alcohol consumption could have interfered with his swallowing ability leading to the aspiration event. The classic triad of cough, dyspnea, and cyanosis is seen in only a small percentage of patients with FBAO resulting in misdiagnosis. [6]. Our patient was initially thought to be having a stroke and a cardiac event based on his right-sided weakness and cardiac arrest, leading to an extensive workup for these conditions. He was found to have moderate to severely dilated RV chamber size, for which a CTA was done identifying a PE. The PE could have been the cause of his initial bloody secretions seen in the ETT and his cardiac arrest. Interestingly, no FB was seen during his initial intubation, during his bronchoscopy, and his emergent re-intubation after his failed initial extubation. Laryngeal edema, soft tissue collapse, and laryngospasm are among the most common causes of upper airway obstruction and can lead to extubation failure [7]. Laryngeal traumatism that can occur in emergency intubations is also an important cause of post-extubation stridor, which was thought to be the cause in our patient [8]. Multiple doses of corticosteroids given 12-24 hours before extubation can be beneficial in patients to prevent stridor after extubation [9]. Due to the sudden decline in our patient’s respiratory status and hypoxia after extubation, he underwent a CT scan of the neck which helped identify abnormal material in his hypopharynx and oropharynx. FB causing complications after tracheal extubation can be seen by dentures or bleeding; however, no case of oropharyngeal FB causing compression of the airway leading to extubation failure has been reported in the literature [1]. We recommend retained FB to be in the differential diagnosis in the causes of immediate hypoxia and extubation failure, even without a history of a witnessed aspiration event as this is an easily correctable cause of extubation failure and can be missed if not considered. Obtaining adequate history and performing a thorough neck and oropharyngeal examination in such a scenario could help identify this easily treatable condition without the need for extensive investigations, thus reducing resource utilization, cost, and radiation exposure. It is easy to miss this as a cause if it is not considered in the differential as no FB was noted by healthcare providers during intubation and bronchoscopy. This is especially important if other conditions can confound the clinical picture such as PE, initial stroke-like presentation, and cardiac arrest seen in our case.

## Conclusions

Acute airway compression can have variable presentations ranging from stroke-like weakness to cardiac arrest. CT of the neck showing abnormal mixed air and soft tissue density in the oropharynx should raise suspicion for retained food in the oropharynx causing airway compression. This can be an important cause of extratracheal compression leading to extubation failure. A thorough direct oropharynx examination should be performed if suspicion of this is raised on the CT scan, and removing the FB can be an easily fixable cause of extubation failure.

## References

[1] Hartley M, Vaughan RS (1993). Problems associated with tracheal extubation. Br J Anaesth.

[2] Couper K, Abu Hassan A, Ohri V (2020). Removal of foreign body airway obstruction: a systematic review of interventions. Resuscitation.

[3] Boo SJ, Kim HU (2018). [Esophageal foreign body: treatment and complications]. Korean J Gastroenterol.

[4] Sehgal IS, Dhooria S, Ram B (2015). Foreign body inhalation in the adult population: experience of 25,998 bronchoscopies and systematic review of the literature. Respir Care.

[5] Saniasiaya J, Mohamad I (2016). Missed hypopharyngeal foreign body: A case report. Egyptian J Ear Nose Throat Allied Sci.

[6] Blanco Ramos M, Botana-Rial M, García-Fontán E, Fernández-Villar A, Gallas Torreira M (2016). Update in the extraction of airway foreign bodies in adults. J Thorac Dis.

[7] Cavallone LF, Vannucci A (2013). Review article: extubation of the difficult airway and extubation failure. Anesth Analg.

[8] Jaber S, Chanques G, Matecki S (2003). Post-extubation stridor in intensive care unit patients. Risk factors evaluation and importance of the cuff-leak test. Intensive Care Med.

[9] Markovitz BP, Randolph AG (2000). Corticosteroids for the prevention and treatment of post-extubation stridor in neonates, children and adults. Cochrane Database Syst Rev.

